# Safety Issues of Long-Term Glucose Load in Patients on Peritoneal Dialysis—A 7-Year Cohort Study

**DOI:** 10.1371/journal.pone.0030337

**Published:** 2012-01-23

**Authors:** Hon-Yen Wu, Kuan-Yu Hung, Tao-Min Huang, Fu-Chang Hu, Yu-Sen Peng, Jenq-Wen Huang, Shuei-Liong Lin, Yung-Ming Chen, Tzong-Shinn Chu, Tun-Jun Tsai, Kwan-Dun Wu

**Affiliations:** 1 Department of Internal Medicine, Far Eastern Memorial Hospital, New Taipei City, Taiwan; 2 Department of Internal Medicine, National Taiwan University Hospital, Taipei, Taiwan; 3 Center of Quality Management, National Taiwan University Hospital, Taipei, Taiwan; 4 National Center of Excellence for General Clinical Trial and Research, National Taiwan University Hospital, Taipei, Taiwan; 5 Department of Internal Medicine, Yun-Lin Branch, National Taiwan University Hospital, Yun-Lin, Taiwan; 6 International Harvard Statistical Consulting Company, Taipei, Taiwan; Children's Hospital Boston/Harvard Medical School, United States of America

## Abstract

**Background:**

Effects of long-term glucose load on peritoneal dialysis (PD) patient safety and outcomes have seldom been reported. This study demonstrates the influence of long-term glucose load on patient and technique survival.

**Methods:**

We surveyed 173 incident PD patients. Long-term glucose load was evaluated by calculating the average dialysate glucose concentration since initiation of PD. Risk factors were assessed by fitting Cox's models with repeatedly measured time-dependent covariates.

**Results:**

We noted that older age, higher glucose concentration, and lower residual renal function (RRF) were significantly associated with a worse patient survival. We found that female gender, absence of diabetes, lower glucose concentration, use of icodextrin, higher serum high density lipoprotein cholesterol, and higher RRF were significantly associated with a better technique survival.

**Conclusions:**

Long-term glucose load predicted mortality and technique failure in chronic PD patients. These findings emphasize the importance of minimizing glucose load in PD patients.

## Introduction

Glucose is the main osmotic agents providing ultrafiltration (UF) to peritoneal dialysis (PD) patients [1], however, a high glucose load may cause peritoneal damage, hyperglycemia, hyperinsulinemia, dyslipidemia, oxidative stress, and increased incidences of metabolic syndrome, as well as cardiovascular diseases (CVD) [2], [3], [4]. We recently reported that higher initial glucose load, defined as the average dialysate glucose concentration prescribed in the first 6 months of PD therapy, predicted a worse PD technique survival [1]. In addition, we also identified that patients with diabetes mellitus (DM), high body mass index (BMI), and low residual renal function (RRF) tend to have a high dialysate glucose load during long-term PD therapy [5]. Regarding long-term patient safety, the accumulative effects from glucose load have rarely been reported. In this retrospective 7-year cohort study of chronic PD patients, we analyze the accumulative effects of long-term glucose load on patient outcomes by applying repeatedly measured time-dependent covariates in survival analysis.

## Methods

### Ethics Statement

The Institutional Review Board of National Taiwan University Hospital approved the retrospective cohort study. Written informed consent was not needed because the study retrospectively collected available medical records in the hospital. The Institutional Review Board specifically granted a waiver for the “no consent needed”.

### Study participants: inclusion and exclusion criteria

202 patients initiated PD therapy at our PD center between September 2001 and January 2006. We excluded those who were younger than 18 years old (n = 4) or had active malignancy (n = 3). Patients who had a PD technique survival shorter than 6 months (n = 10) or had undergone PD previously (n = 12) were also excluded. A total of 173 end-stage renal disease (ESRD) patients were enrolled in our study and were followed until February 2008. The mean follow-up period was 42.0±17.2 (range 6–78) months.

We reviewed clinical data of each patient, including age, gender, and annual data of comorbid diseases, body height, body weight, RRF, peritoneal transport characteristics and solute clearance, cardiothoracic ratio (CTR), and blood laboratory exams. Comorbid diseases included DM, CVD, and chronic hepatic diseases. Patients with a history of stroke, coronary or peripheral artery diseases, or left ventricular ejection fraction less than 30% were considered to have CVD [5], [6]. Chronic hepatic diseases included liver cirrhosis and chronic viral hepatitis [7].

### Definitions of patient survival and PD technique survival

The primary outcome of this study was actuarial patient survival and the secondary outcome was PD technique survival. In the analysis of patient survival, the causes of drop-out were classified as death, renal transplantation, and hospital transfer. Because long-term survival is much better for renal transplant recipients than patients who remain on dialysis [8], renal transplantation, as well as hospital transfer, were considered as censoring events in the analysis of patient survival. In the analysis of PD technique survival, the reasons for terminating PD were categorized as hospital transfer, renal transplantation, death, and switching to hemodialysis (HD). Among them, events of switching to HD were recognized as PD technique failure, while the others were regarded as censoring events.

### PD regimens and calculation of glucose load

The PD regimen and modality for each patient were evaluated and prescribed in our PD unit during monthly follow-up. We reviewed the detailed PD regimen of each patient. Our evaluations for glucose exposure included glucose weight and glucose load. Glucose weight was defined as the sum of the products of glucose concentration and the volume for each exchange of glucose solution (Dianeal®; Baxter Healthcare Corporation, Deerfield, IL, U.S.A.) over a time period [9], whereas glucose load was calculated as the average dialysate glucose concentration (i.e., [total glucose weight]/[total volume of glucose solution]) over a time period [10], [11]. The administered volume of icodextrin solution (Extraneal®; Baxter Healthcare) and amino acid solution (Nutrineal®; Baxter Healthcare) for each patient was also evaluated.

### Laboratory examinations and peritoneal dialysis assessments

The blood for laboratory exams was drawn after patients had fasted for at least 8 hours. We evaluated dialysate-to-plasma ratio of creatinine (D/P Cre) and UF volume by the peritoneal equilibration test (PET) [12]. We measured peritoneal solute clearance by weekly peritoneal Kt/V, evaluated RRF based on weekly renal Kt/V, and estimated protein intake by normalized protein nitrogen appearance (nPNA) [13]. All biochemical and hematological tests were performed with automatic analyzers.

### Statistical analyses

Data are expressed as mean ± standard deviation (SD) or frequency and percentage, unless otherwise indicated. As descriptive analysis, univariate analyses were performed by using two-sample *t* tests, one-way analysis of variance (ANOVA), Pearson's chi-square test, or Fisher's exact test, as appropriate. In multivariate analysis, the risk factors of mortality and PD technique failure were assessed by fitting two different Cox's models with repeatedly measured time-dependent covariates using the counting process approach. The repeatedly measured time-dependent covariates included body weight, comorbidity status, dialysate prescription, PD modality, laboratory blood exams, peritoneal transport, estimated protein intake, and solute clearance by the peritoneum and kidneys. Among them, each subject's dialysate prescription was represented by (1) the accumulative glucose exposure and (2) the accumulative volumes of icodextrin solution and amino acid solution, from the time of PD initiation to each time at which a PD technique failure or death occurred in the Cox's models for PD technique failure or mortality.

Next, the predictors of each year's annual average dialysate glucose concentration over the 6-year follow-up period were examined. Since the missing data in the repeated-measurements of glucose concentration at later times was most likely due to PD technique failure, 6 separate multiple linear regression models were fitted to the data of the subjects who were still at risk in the beginning of each year.

To assure the quality of the analysis results, we applied basic model-fitting techniques, including stepwise variable selection, goodness-of-fit assessment, and regression diagnostics (e.g., residual analysis, detection of influential cases, and checks for multicollinearity), in our regression analysis. The stepwise variable selection procedure (with iterations between the forward and backward steps) was applied to obtain the candidate final regression model. All the univariate significant and non-significant relevant covariates and some of their interactions were put on the variable list to be selected. The significance levels for entry and for stay were set to 0.15 or larger. Since the statistical testing at each step of the stepwise variable selection procedure was conditioning on the other covariates in the regression model, the concern about multiple analyses is minor. We used the variance inflation factor to detect the potential multicollinearity problem. A grid search method was applied to discover appropriate cut-off points for representing nonlinear effects of some continuous covariates. A two-sided *P* value ≤0.05 was considered statistically significant. The statistical analyses were conducted using SAS software, version 9.1.3 (SAS Institute Inc., Cary, NC, USA).

## Results

### Patient characteristics

There were 87 men and 86 women enrolled in our study with a mean age of 54.6±15.6 years at PD initiation. Patients with longer technique survivals had higher BMI, higher serum levels of total cholesterol, triglyceride (TG), albumin, and creatinine (Table 1). We noted a trend of a decrease in RRF with increasing peritoneal Kt/V among patients who had more prolonged PD therapy. Moreover, these patients were prescribed with higher doses of icodextrin solution and showed a higher UF volume in later years (Table 1). Patients with longer overall survivals had higher BMI and higher serum levels of total cholesterol, TG, albumin, and creatinine (Table 2). During the study period, 4 cases of new-onset DM were diagnosed, and we considered the change in DM status as a repeatedly measured time-dependent covariate in the regression analysis.

**Table 1:** Demographic and clinical data of patients who remained on PD at the beginning of each year.

	Treatment Year																	
Variable	First year			Second year			Third year			Fourth year			Fifth year			Sixth year		
Patient number	173			151			133			86			49			13		
Gender(M∶F)	87	:	86	75	:	76	64	:	69	37	:	49	22	:	27	5	:	8
Baseline age (years)	54.6	±	15.6	53.3	±	14.7	53.2	±	14.0	51.0	±	12.0	50.8	±	9.2	52.0	±	8.6
Glucose concentration (%)*	1.81	±	0.26	1.79	±	0.33	1.80	±	0.39	1.81	±	0.42	1.76	±	0.71	1.90	±	0.68
Glucose weight (Kg/month)**	4.9	±	1.5	4.9	±	1.7	5.0	±	1.7	5.1	±	1.9	4.8	±	2.3	4.3	±	1.9
Icodextrin solution (L/month)**, ξ	3.6	±	14.1	7.2	±	18.9	12.5	±	24.9	9.8	±	21.4	11.5	±	21.1	25.5	±	25.8
Amino acid solution (L/month)**	1.3	±	5.4	2.2	±	8.0	2.0	±	8.7	0.7	±	3.2	0.8	±	2.7	0.0	±	0.0
Body mass index (Kg/m^2^)ξ	21.6	±	3.2	22.8	±	3.4	23.0	±	3.3	22.9	±	2.8	23.0	±	2.4	22.6	±	2.8
Comorbid diseases																		
Diabetes mellitus	37	(21.4)		29	(19.2)		22	(16.5)		10	(11.6)		5	(10.2)		3	(23.1)	
Cardiovascular diseases	64	(37.0)		52	(34.4)		41	(30.8)		24	(27.9)		10	(20.4)		5	(38.5)	
D/P Cre	0.63	±	0.10	0.62	±	0.10	0.61	±	0.10	0.61	±	0.10	0.62	±	0.10	0.65	±	0.07
Ultrafiltration volume (ml)***, ξ	278	±	187	333	±	173	376	±	332	347	±	221	441	±	265	456	±	154
PD modality (CAPD∶APD)	144	:	29	121	:	30	97	:	36	61	:	25	36	:	13	12	:	1
Weekly peritoneal Kt/Vξ	1.71	±	0.36	1.85	±	0.33	1.94	±	0.27	1.96	±	0.28	2.09	±	0.29	2.09	±	0.33
Weekly renal Kt/Vξ	0.60	±	0.44	0.37	±	0.36	0.25	±	0.31	0.21	±	0.30	0.10	±	0.19	0.04	±	0.15
Daily urine output (ml)ξ	742	±	558	580	±	555	440	±	568	365	±	514	190	±	346	130	±	413
nPNA (g/Kg/d)	1.10	±	0.25	1.09	±	0.23	1.08	±	0.23	1.05	±	0.22	1.04	±	0.20	0.98	±	0.24
Cardiothoracic ratio (%)	51.4	±	7.5	50.1	±	6.7	49.9	±	6.7	49.6	±	6.6	50.5	±	6.2	49.5	±	10.8
Hemoglobin (g/dL)	9.9	±	1.6	9.8	±	1.3	9.8	±	1.4	9.9	±	1.3	9.8	±	1.8	10.0	±	1.5
Blood biochemistry																		
Total cholesterol (mg/dL)ξ	201	±	52	222	±	52	217	±	52	214	±	47	204	±	60	218	±	83
Triglyceride (mg/dL)ξ	156	±	111	213	±	151	218	±	171	221	±	166	214	±	155	313	±	431
HDL (mg/dL)	44.0	±	13.6	41.7	±	11.5	41.5	±	15.6	40.3	±	8.9	40.5	±	9.3	40.8	±	9.2
Fasting glucose (mg/dL)	111	±	43	111	±	39	103	±	43	100	±	29	107	±	28	99	±	18
Albumin (g/dL)ξ	3.8	±	0.5	4.0	±	0.4	4.0	±	0.4	4.0	±	0.3	4.1	±	0.4	4.0	±	0.2
Urea nitrogen (mg/dL)	65.5	±	22.2	62.8	±	16.0	63.3	±	16.4	62.9	±	15.1	64.1	±	16.9	63.1	±	10.8
Creatinine (mg/dL)ξ	9.2	±	3.0	11.0	±	2.9	11.6	±	2.9	11.5	±	3.3	12.3	±	2.5	11.3	±	2.3

NOTE. Data are expressed as mean ± SD. Conversion factors for units: hemoglobin in g/dL to g/L, ×10; cholesterol and HDL in mg/dL to mmol/L, ×0.02586; triglyceride in mg/dL to mmol/L, ×0.01129; glucose in mg/dL to mmol/L, ×0.05551; albumin in g/dL to to g/L, ×10; urea nitrogen in mg/dL to mmol/L, ×0.357; creatinine in mg/dL to µmol/L, ×88.4.

Abbreviations: D/P, dialysate-to-plasma; CAPD, continuous ambulatory peritoneal dialysis; APD, automated peritoneal dialysis; nPNA, normalized protein nitrogen appearance; HDL, high-density lipoprotein cholesterol.

*The annual average dialysate glucose concentration prescribed within each year.

**Obtained from all the PD solution prescribed within each year.

***Obtained from the peritoneal equilibration test.

ξ *P*<0.05 for the statistical testing between years.

**Table 2:** Demographic and clinical data of patients who remained alive at the beginning of each year.

	Treatment Year																	
Variable	First year			Second year			Third year			Fourth year			Fifth year			Sixth year		
Patient number	173			159			147			99			58			21		
Gender(M∶F)	87	:	86	81	:	78	75	:	72	46	:	53	29	:	29	9	:	12
Baseline age (years)	54.6	±	15.6	53.9	±	15.2	53.3	±	14.2	51.0	±	12.7	49.9	±	11.9	49.3	±	9.1
Glucose concentration (%)*, ξ	1.81	±	0.26	1.70	±	0.51	1.63	±	0.65	1.57	±	0.73	1.48	±	0.91	1.17	±	1.08
Glucose weight (Kg/month)**, ξ	4.9	±	1.5	4.7	±	2.0	4.5	±	2.2	4.5	±	2.5	4.1	±	2.8	2.7	±	2.6
Icodextrin solution (L/month)**, ξ	3.6	±	14.1	6.8	±	18.5	11.5	±	23.9	8.5	±	20.2	9.7	±	19.8	15.8	±	23.7
Body mass index (Kg/m^2^)ξ	21.6	±	3.2	22.7	±	3.4	23.1	±	3.4	22.9	±	2.9	22.8	±	2.5	23.2	±	3.3
Comorbid diseases																		
Diabetes mellitus	37	(21.4)		32	(20.1)		29	(19.7)		13	(13.1)		6	(10.3)		4	(19.0)	
Cardiovascular diseases	64	(37.0)		56	(35.2)		47	(32.0)		26	(26.3)		11	(19.0)		6	(28.6)	
Weekly renal Kt/Vξ	0.60	±	0.44	0.37	±	0.37	0.25	±	0.30	0.21	±	0.29	0.10	±	0.19	0.03	±	0.13
Hemoglobin (g/dL)	9.9	±	1.6	9.75	±	1.29	9.86	±	1.42	9.88	±	1.51	9.82	±	2.00	10.38	±	1.94
Blood biochemistry																		
Total cholesterol (mg/dL)ξ	201	±	52	221	±	52	215	±	52	211	±	48	200	±	59	212	±	80
Triglyceride (mg/dL)ξ	156	±	111	213	±	149	212	±	166	218	±	166	206	±	149	282	±	372
Fasting glucose (mg/dL)	111	±	43	113	±	42	104	±	43	100	±	29	106	±	27	101	±	20
Albumin (g/dL)ξ	3.8	±	0.5	3.9	±	0.4	4.0	±	0.4	4.0	±	0.4	4.1	±	0.4	4.0	±	0.4
Urea nitrogen (mg/dL)	65.5	±	22.2	62.8	±	16.1	63.8	±	17.3	62.6	±	15.7	63.6	±	17.6	60.6	±	13.4
Creatinine (mg/dL)ξ	9.2	±	3.0	10.9	±	3.0	11.5	±	2.9	11.5	±	3.3	12.2	±	2.5	10.5	±	3.0

NOTE. Data are expressed as mean ± S.D. Conversion factors for units: hemoglobin in g/dL to g/L, ×10; cholesterol in mg/dL to mmol/L, ×0.02586; triglyceride in mg/dL to mmol/L, ×0.01129; glucose in mg/dL to mmol/L, ×0.05551; albumin in g/dL to to g/L, ×10; urea nitrogen in mg/dL to mmol/L, ×0.357; creatinine in mg/dL to µmol/L, ×88.4.

*The annual average dialysate glucose concentration prescribed within each year.

**Obtained from all the PD solution prescribed within each year.

ξ *P*<0.05 for the statistical testing between years.

### Patient survival analysis

**Figure 1:**
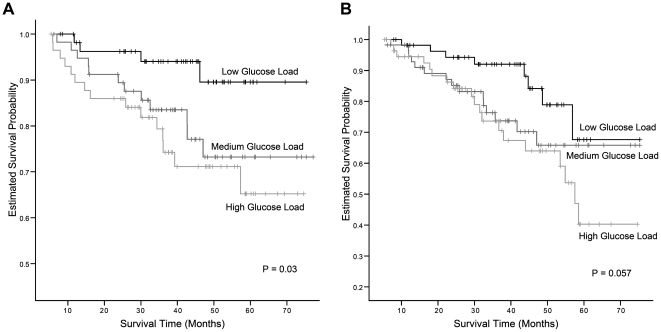
Cumulative survival curves for (A) patient survival and (B) technique survival. All subjects were divided into tertiles (low, medium, or high glucose load group) according to the average dialysate glucose concentration administered since PD initiation. Survival curves are constructed by the Kaplan-Meier method and compared by the log-rank test. Subjects with higher glucose load showed worse patient and technique survivals.

By the end of the follow-up, there were 60 dropouts, including 31 deaths, 20 renal transplantations, and 9 patients transferred to other hospitals. The major causes of death included infectious diseases (64.5%) and CVD (12.9%). The annual patient survival rates by using the Kaplan-Meier method were 94.7%, 90.5%, 85.5%, 78.0%, and 75.5% at the beginning of year-2, year-3, year-4, year-5, and year-6, respectively. Kaplan-Meier survival curves showed a significant worse patient survival in the subjects with higher glucose load (Figure 1A, *P* = 0.03). The mean patient survival time was 65.5±1.9 months. By fitting Cox's models with repeatedly measured time-dependent covariates, we found that older age, higher glucose load, lower RRF, higher blood urea nitrogen (BUN), and lower serum creatinine were significantly associated with a worse patient survival (Table 3). The presence of CVD had a borderline significant influence on patient survival (Table 3), and the positive association between the presence of CVD and DM was highly significant (odds ratio = 7.224, *P*<0.001). This could explain why only the presence of CVD, but not also DM, was significant in patient survival analysis.

**Table 3:** Multivariate analyses of the risk factors for patient and technique survival using Cox's models with repeatedly measured time-dependent covariates.

	Parameter		Standard			Hazard	95% Confidence		
Covariate	Estimate (B)		Error	*P* Value		Ratio	Interval		
**Patient Survival** 1 **:**									
Baseline age (years)		0.07	0.02	<	0.001	1.07	1.03	-	1.11
Cardiovascular diseases		0.99	0.54		0.07	2.68	0.92	-	7.76
Glucose concentration (%)*		1.83	0.81		0.02	6.23	1.26	-	30.74
Weekly renal Kt/V	-	3.18	1.04		0.002	0.04	0.01	-	0.32
Blood urea nitrogen (mg/dL)		0.02	0.01		0.01	1.02	1.004	-	1.04
Creatinine (mg/dL)	-	0.40	0.09	<	0.001	0.67	0.56	-	0.80
**Technique Survival** 2 **:**									
Male gender		1.39	0.40	<	0.001	4.03	1.85	-	8.75
Diabetes mellitus		1.53	0.41	<	0.001	4.64	2.07	-	10.38
Glucose concentration (%)*		2.25	0.90		0.01	9.46	1.62	-	55.35
Glucose weight (Kg/month)*	-	0.64	0.17	<	0.001	0.53	0.38	-	0.73
Icodextrin solution (L/month)*	-	0.02	0.01		0.03	0.98	0.96	-	0.997
Weekly renal Kt/V	-	2.03	0.72	<	0.01	0.13	0.03	-	0.53
1.4 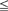 Weekly peritoneal Kt/V <1.7	-	1.53	0.63		0.02	0.22	0.06	-	0.74
HDL (mg/dL)	-	0.05	0.02		0.02	0.95	0.92	-	0.99

NOTES. Conversion factors for units: HDL in mg/dL to mmol/L, ×0.02586; urea nitrogen in mg/dL to mmol/L, ×0.357; creatinine in mg/dL to µmol/L, ×88.4.

Abbreviations: HDL, high-density lipoprotein cholesterol.

1 Cox's model with 4 repeatedly measured time-dependent covariates (glucose concentration, weekly renal Kt/V, blood urea nitrogen, and creatinine), adjusted generalized *R* ^2^ = 0.58.

2 Cox's model with 7 repeatedly measured time-dependent covariates (diabetes mellitus, glucose concentration, glucose weight, icodextrin solution, weekly renal Kt/V, weekly peritoneal Kt/V, and HDL), adjusted generalized *R* ^2^ = 0.31.

*Each subject's dialysate prescription was represented by (1) the accumulative glucose exposure and (2) the accumulative volumes of icodextrin solution, from the time of PD initiation to each time at which a PD technique failure or death occurred in the Cox's models for PD technique failure or mortality.

### PD technique survival analysis

By the end of the follow-up, there were 42 PD technique failures, including peritonitis (42.9%), UF failure (21.4%), exit site infection (19.0%), and inadequate solute clearance (14.3%). Events of random censorings included 20 renal transplantations, 3 hospital transfers, and 19 deaths (mainly due to infectious diseases and CVD). The annual PD technique survival rates by using the Kaplan-Meier method were 95.7%, 88.5%, 79.8%, 71.6%, and 56.4% at the beginning of year-2, year-3, year-4, year-5, and year-6, respectively. Kaplan-Meier survival curves showed a borderline significant worse technique survival in the subjects with higher glucose load (Figure 1B, *P* = 0.057). The mean technique survival time was 58.8±2.1 months. By fitting Cox's models with repeatedly measured time-dependent covariates, we found that male gender, the presence of DM, higher glucose load, lower RRF, and lower serum high-density lipoprotein (HDL) were significantly associated with a worse technique survival (Table 3). Higher glucose weight and the use of icodextrin solution were significantly associated with a better technique survival (Table 3). By Pearson's correlation analysis, a higher dialysate glucose weight was significantly correlated with a higher volume of glucose solution (*r* = 0.861, *P*<0.001), and the correlation between the volume of glucose solution and average dialysate glucose concentration was much lower (*r* = 0.274, *P*<0.001). Higher peritoneal Kt/V (without adding weekly renal Kt/V) was not persistently associated with a better technique survival. Further analysis of discretized weekly peritoneal Kt/V (without adding weekly renal Kt/V) with different cut-off values revealed that the range between 1.4 and 1.7 was associated with the best technique survival (Table 3).

### Factors determining annual glucose load

**Figure 2:**
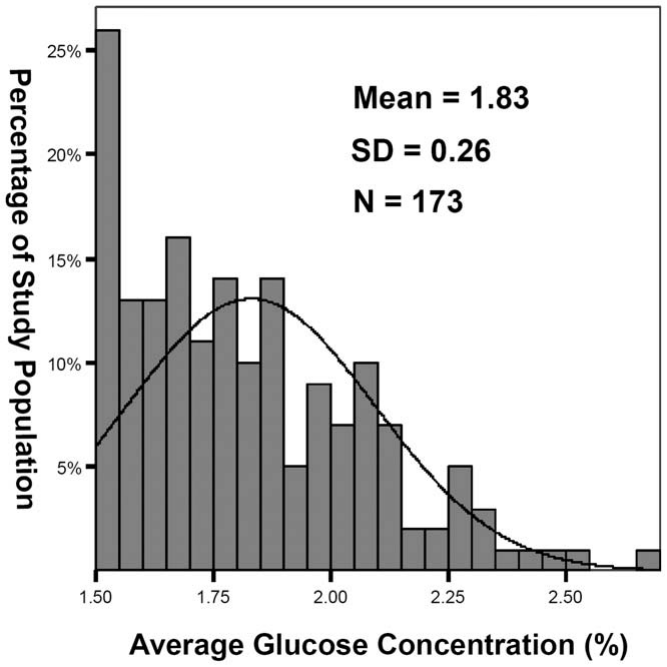
Frequency distribution of average dialysate glucose concentration in the study population. Average dialysate glucose concentration of each subject was calculated as: [total glucose weight]/[total volume of glucose solution] administered since initiation of PD.

Figure 2 shows the distributions of glucose load in our study population. Considering the gradual increase in the number of dropouts due to technique failure, we performed multiple linear regressions to analyze the predictors for annual glucose load (average dialysate glucose concentration) among patients who remained on PD at the beginning of that year. We applied PD year-specific multiple linear regression models, using the stepwise variable selection method, to analyze the effect of gender, age, BMI, comorbid diseases, PD modality, icodextrin solution, amino acid solution, peritoneal characteristics and solute clearance, RRF, CTR, and results of blood tests. As shown in Table 4, the presence of DM, higher BMI, lower weekly renal Kt/V, younger age, and the use of icodextrin solution were significantly correlated with higher glucose load during most of the years of the study period. We also observed that the modality of automated PD (APD), higher CTR, lower peritoneal UF volume, lower serum albumin, and lower BUN were significantly correlated with higher glucose load in some years during the study period (Table 4).

**Table 4:** Multiple linear regression analyses of predictors associated with annual average dialysate glucose concentration administered within each year^a^.

	Treatment Year																	
Covariate	First year^1^			Second year^2^			Third year^3^			Fourth year^4^			Fifth year^5^			Sixth year^6^		
Baseline age (years)	—			−0.004	±	0.002*	−0.003	±	0.002*	−0.011	±	0.003**	−0.022	±	0.005**	−0.020	±	0.007**
Diabetes mellitus	0.226	±	0.040**	0.177	±	0.056**	0.266	±	0.064**	0.186	±	0.093*	0.599	±	0.136**	0.288	±	0.160§
Body mass index (Kg/m^2^)	0.020	±	0.005**	0.028	±	0.006**	0.028	±	0.007**	0.036	±	0.012**	—			—		
Cardiothoracic ratio (%)	—			0.783	±	0.336*	—			1.328	±	0.493**	—			—		
Weekly renal Kt/V	−0.090	±	0.036**	−0.174	±	0.055**	−0.355	±	0.071**	−0.232	±	0.096*	—			—		
Icodextrin solution (kl/month)^b^	0.033	±	0.008**	0.025	±	0.008**	—			—			0.055	±	0.026*	0.107	±	0.019**
Ultrafiltration volume (L)^c^	—			—			−0.418	±	0.128**	−0.479	±	0.139**	—			—		
Automated peritoneal dialysis	0.081	±	0.041*	—			—			—			—			—		
Albumin (g/dL)	−0.079	±	0.031**	—			—			—			—			—		
Blood urea nitrogen (mg/dL)	—			—			—			−0.004	±	0.002*	—			—		

NOTE. Conversion factors for units: albumin in g/dL to to g/L, ×10; urea nitrogen in mg/dL to mmol/L, ×0.357.

a The numbers listed in the table are the least squares estimates of regression coefficients ± estimates of the corresponding standard errors.

b Obtained from all the PD solution prescribed within each year.

c Obtained from the peritoneal equilibration test.

*p≤0.05.

**p≤0.01.

§ 0.05<*P*<0.1.

1 Linear regression model: n = 173, *R* ^2^ = 0.4101.

2 Linear regression model: n = 150, *R* ^2^ = 0.3751.

3 Linear regression model: n = 129, *R* ^2^ = 0.4534.

4 Linear regression model: n = 84, *R* ^2^ = 0.4466.

5 Linear regression model: n = 44, *R* ^2^ = 0.5664.

6 Linear regression model: n = 22, *R* ^2^ = 0.7284.

## Discussion

This is the first detailed survival analysis describing the long-term effects of glucose load on PD outcomes by applying the repeatedly measured time-dependent method. In this 7-year cohort, patients with higher long-term glucose load were significantly associated with worse actuarial patient survival and PD technique survival (Table 3). In addition, the prescription of icodextrin solution was significantly associated with a better PD technique survival (Table 3). Furthermore, younger age was one of the main risk factors for high glucose load in long-term PD (Table 4).

Previous studies on PD patients have shown that the actual glucose absorption by the peritoneum can be precisely predicted from the average glucose concentration in the dialysate inflow, which offers an easy way to evaluate glucose uptake in long-term PD patients [11]. In the present study, we demonstrated further that a higher chronic glucose load was significantly associated with a worse patient survival, as well as a worse technique survival (Table 3). This result is compatible with our previous report regarding the harmful effects of the average dialysate glucose concentration in the initial 6 months after PD commencement [1]. A higher average dialysate glucose concentration is more directly related with the consumption of high glucose-containing PD solution, implying more glucose uptake, poor fluid control, and more peritoneal damage [3], [5], [11]. In contrast, prescription of icodextrin solution is associated with a better PD technique survival, which may result from the beneficial effects of icodextrin in improving fluid control and metabolic abnormalities [2], [3], [14].

Because of the regulations of the health insurance system in Taiwan, icodextrin solution is prescribed to patients who require 2.5% or 4.25% dextrose solution in more than half of their daily dialysate volume. This leads to the association between the use of icodextrin solution and higher average dialysate glucose concentration (Table 4). PD patients require dialysate with higher glucose concentration if their fluid control is inadequate, thus higher CTR and lower peritoneal UF volume have some associations with higher glucose load (Table 4). Daily oral intake in elderly PD patients may decrease due to diminished appetite, dental problems, constipation, and increased intra-abdominal pressure by the dialysate [15]. This may result in less use of PD solution with high glucose concentration in elderly patients (Table 4).

PD patients with lower levels of serum creatinine or higher levels of BUN were significantly associated with higher mortality (Table 3). Serum creatinine is highly correlated with lean body mass and nutritional condition in patients on chronic dialysis therapy, and the association between low serum creatinine and high mortality has been reported in previous studies [16]. A “U”-shaped pattern of the influence of BUN on dialysis patients has been reported, with higher mortality in patients with BUN over 110 mg/dL or below 60 mg/dL. This phenomenon might result from malnutrition in low BUN groups and under dialysis in high BUN groups [16], [17]. This explains the association between higher mortality and lower levels of serum creatinine or higher levels of BUN (Table 3).

There is a significant correlation between high glucose weight and high dialysate volume (*r* = 0.861), hence, the beneficial effect of total glucose weight (Table 3) might reflect a better peritoneal solute clearance. We noted that patients with weekly peritoneal Kt/V (without adding weekly renal Kt/V) between 1.4 and 1.7 had significantly better technique survivals (Table 3). Similar results were also reported by Lo et al. They noted a “V” shaped curve of mortality risk in female anuric PD patients, with the best survival rates among those with weekly peritoneal Kt/V between 1.67 and 1.86 [18]. According to the guidelines for solute removal in chronic PD, the weekly total Kt/V (peritoneal Kt/V plus renal Kt/V) should be at least 1.7 or above [19]. However, there are no additional beneficial effects on patient outcomes when increasing the weekly total Kt/V further, and potential adverse effects of increasing PD dose include increased intraperitoneal pressure, failure to increase clearance of middle molecules, and increased exposure to glucose [18].

This study certainly has a few limitations. First, this is a retrospective cohort analysis, and the causal relationships between variables and outcomes could be influenced by confounding factors. Even though we have controlled for several important covariates, the possibility of residual confounding still remains. Second, the patient number of our study cohort is slightly limited; however, the statistical power for testing important hypotheses should be enough in Cox's models using repeatedly measured time-dependent covariates. Third, it is difficult to clearly distinguish the study population into either continuous ambulatory PD (CAPD) or APD groups. Patients initiated PD with the modality of CAPD, and may have shifted between APD and CAPD according to medical advice and individual family facilities or convenience. We tried to minimize this limitation by applying PD modality as a repeatedly measured time-dependent covariate in the regression analysis. Fourth, although our PD center belongs to a tertiary referral university hospital with patients from the whole country, this is a single-center study and the applicability of our findings to general PD population is still limited. To resolve these limitations, a prospective, large scale, multi-center randomized trial comparing different PD regimens is necessary.

In conclusion, our study demonstrates that long-term glucose load predicts mortality and technique failure in chronic PD patients, and the use of icodextrin is associated with a better technique survival.
